# Comparative Analysis of Primary and Revision Single-Level Lumbar Fusion Surgeries: Predictors, Outcomes, and Clinical Implications Using Big Data

**DOI:** 10.3390/jcm14030723

**Published:** 2025-01-23

**Authors:** Assil Mahamid, Fairoz Jayyusi, Marah Hodruj, Amr Mansour, Dan Fishman, Eyal Behrbalk

**Affiliations:** 1 Department of Orthopedics, Hillel Yaffe Medical Center, Hadera 3820302, Israel; 2 Rappaport Faculty of Medicine, Technion University Hospital (Israel Institute of Technology), Haifa 3200003, Israel; 3 Bnai-Zion Medical Center, Haifa 3339419, Israel

**Keywords:** inpatient outcomes, lumbar fusion, National Inpatient Sample, revision fusion, healthcare utilization

## Abstract

**Background/Objectives:** The etiology of lumbar spine revision surgery is multifactorial, involving mechanical, biological, and clinical factors that challenge sustained spinal stability. Comparative analysis reveals significantly higher complication rates, prolonged hospital stays, and increased costs for revision surgeries compared to primary fusions, despite low mortality rates. Leveraging a comprehensive dataset of 456,750 patients, this study identifies predictors of revision surgery and provides actionable insights to enhance patient outcomes and optimize healthcare resource allocation. **Methods:** A total of 456,750 patients registered in the National Inpatient Sample (NIS) database from 2016 to 2019 were identified as having undergone single-level lumbar fusion surgery (primary fusion: 99.5%; revision fusion: 0.5%). Multivariable logistic regression models adjusted for patient demographics, clinical comorbidities, and hospital characteristics were constructed to evaluate clinical outcomes and postoperative complications. **Results:** Patients undergoing revision lumbar fusion surgery were significantly younger compared to those undergoing primary fusion procedures (53.92 ± 20.65 vs. 61.87 ± 12.32 years, *p* < 0.001); among the entire cohort, 56.4% were women. Compared with patients undergoing primary lumbar fusion, those undergoing revision fusion surgery were significantly more likely to experience surgical site infections (odds ratio [OR] 27.10; 95% confidence interval [95% CI] 17.12–42.90; *p* < 0.001), urinary tract infections (OR 2.15; 95% CI 1.39–3.33; *p* < 0.001), and prolonged length of stay (OR 1.53; 95% CI 1.24–1.89; *p* < 0.001). Revision surgery patients had significantly lower odds of incurring high-end hospital charges (OR 0.65; 95% CI 0.51–0.83; *p* < 0.001). Other complications, including respiratory complications, dural tears, thromboembolic events, and acute renal failure, showed no statistically significant differences between the two groups. In-hospital mortality rates were low and did not differ significantly between groups (revision: 0.2% vs. primary: 0.1%, OR 3.29; 95% CI 0.45–23.84; *p* = 0.23). **Conclusions:** Patients undergoing revision lumbar fusion surgeries face significantly higher risks of surgical site infections, urinary tract infections, and prolonged hospital stays compared to primary fusion procedures. These findings highlight the need for targeted interventions to improve perioperative management and reduce complications in revision lumbar fusion surgery.

## 1. Introduction

The etiology of revision lumbar fusion (rLF) spine surgery following primary lumbar fusion (pLF) surgery is complex and multifactorial, reflecting the inherent difficulties in maintaining long-term spinal stability and function. Contributing factors include mechanical issues such as implant failure and pseudarthrosis, biological factors like adjacent segment disease (ASD) and infections, and clinical challenges such as deformity progression, recurrent stenosis, and neurological impairments [1,2,3,4,5]. Evidence highlights that patients undergoing rLF procedures experience significantly higher complication rates compared to pLF. Revision surgeries have been associated with increased risks of unfavorable discharge outcomes, prolonged hospitalization, elevated healthcare expenditures, and higher incidences of neurologic complications, deep venous thrombosis, pulmonary embolism, wound infections, and gastrointestinal complications [6]. Similarly, elevated rates of reoperations and poorer clinical outcomes have been reported, though hospital readmission rates appear similar between rLF and pLF groups [3]. Complications associated with rLF procedures for adult spinal deformities also result in extended hospitalizations when compared to primary surgeries, even after adjusting for baseline comorbidities [7]. However, short-term complication rates between primary and revision posterior lumbar fusions do not significantly differ, though revision surgeries necessitate more frequent blood transfusion [8]. The prevalence of rLF surgery is highly variable, ranging from 6% to 24% of all lumbar fusion (LF) cases. Prevalence rates as high as 23.6% and as low as 6% have been reported [3,9].

Despite this variability, the data underscore the significant burden of revision procedures within spinal surgery. Mortality rates following rLF surgeries remain low but vary depending on patient demographics and comorbidities. No significant differences in in-hospital mortality rates have been observed between primary and revision surgeries for adult spinal deformities, with comparable outcomes in both cohorts [7]. Nevertheless, the increased risk of complications associated with revision surgeries may contribute indirectly to elevated mortality risks.

This study leverages a comprehensive dataset of 456,750 patients recorded in the National Inpatient Sample (NIS) database who underwent single-level lumbar fusion surgery between 2016 and 2019. Our primary objective is to advance the current discourse surrounding the efficacy of these procedures by deepening understanding of their practical implications, advantages, and limitations. Through this investigation, we aim to provide valuable insights that will inform future research and clinical decision-making, thereby enhancing patient-centered care and optimizing the allocation of healthcare resources.

## 2. Methods

### 2.1. Data Source

The NIS database formulated by the Agency for Healthcare Research and Quality (Rockville, Maryland, USA) for the Healthcare Cost and Utilization Project [HCUP] was the data source used for the present study. The NIS captures approximately 20% of inpatient stays from HCUP-associated hospitals, representing approximately 7 million unweighted admissions annually. Using discharge sample weights provided by the NIS, these data can be extrapolated to generate national estimates. The dataset analyzed spans 1 January 2016 to 31 December 2019, representing the latest available information within the NIS system at the time of this study. Each dataset entry, referred to as a “case”, encapsulated a group of 5 patients, meticulously matched on general parameters. This numerical approach reflects the NIS discharge weighting methodology, where each case corresponds to five patients. A total of 91,350 cases involving LF surgery were analyzed, representing 456,750 patients. All of these 456,750 patients underwent one-level minimal invasive LF surgery, with 2470 of them undergoing revision surgery as identified by the ICD10 procedure codes (Table 1). This subset represents 0.54% of the total LF surgery patients. The study received approval from the relevant institutional review board, and the requirement for informed consent was waived due to the de-identified nature of the data sourced from the NIS.

### 2.2. Cohort Definition and Selection Criteria

The NIS database was queried for the years 2016–2019 to identify adult patients (aged > 18 years) who underwent single-level LF surgery, categorized as either primary (first, pLF) or secondary (revision, rLF) procedures

### 2.3. Outcome Variables (End Points)

The primary objective of this study was to identify predictors of revision surgery by analyzing factors associated with the need for reoperation. Additionally, primary outcome variables of interest included inpatient outcomes and complications after primary and revision LF surgeries. The primary endpoints were inpatient mortality, length of stay, hospital charges, and inpatient postoperative outcomes, including neurologic complications (dural tears, CSF leak, nerve root injury), venous thromboembolism (deep venous thrombosis [DVT] and pulmonary embolism [PE]), surgical site infection, cardiac complications (myocardial infarction, cardiac arrest), respiratory complications (pneumonia, respiratory failure), and acute renal failure. Continuous outcome variables, length of stay (LOS), and hospital charges were dichotomized. Patients with LOS above the 75th percentile of total inpatient duration, from admission until discharge, were designated as having a “prolonged LOS”. Similarly, patients billed with charges greater than the 75th percentile of the mean charges for inpatient stay were classified as having “high-end hospital charges”.

### 2.4. Exposure Variables

The patient-level characteristics included age, sex, race, primary payer, and comorbidities. The latter included heart disease (congestive heart failure and hypertension), chronic obstructive pulmonary disease, chronic renal disease, type 2 diabetes mellitus, alcohol abuse, Parkinson’s disease, Alzheimer’s disease, mental disorders, dyslipidemia, chronic anemia, and obstructive sleep apnea (Table 2). To prevent unstable coefficients in the regression models, minute categories of patient demographics were coalesced. To this effect, patients designated as “Native American” in race and with “no charge” or “self-pay” were denominated to “other race” and “other” payer, respectively. The hospital characteristics comprised hospital bed size, location and academic status, and geographical region.

Demographic, clinical, and hospital characteristics across patients undergoing primary and revision lumbar fusion (LF) surgeries were compared using the Pearson χ2 test for categorical covariates and independent samples *t*-test for continuous variables. Descriptive statistics are reported as numbers and percentages for categorical variables, whereas continuous variables are depicted as mean ± SD or median and interquartile range as appropriate. Hospital charges were rounded to the nearest whole number.

Missing data analysis revealed that race had the highest proportion of missing values (5.52%), followed by total charge (0.36%), elective (0.21%), and primary payer (0.12%). Other variables, including mortality and female gender, had minimal missing data (0.03% and 0.02%, respectively). Missing data were handled using multivariate single imputation to maintain the analytical sample and minimize bias.

The analysis employed survey-weighted multivariable logistic regression models for two purposes. First, to identify predictors of revision versus primary surgery, where the dependent variable was surgery type. Second, to examine the associations between surgery type (revision vs. primary lumbar fusion) and four primary outcomes: prolonged length of stay, high-end hospital charges, surgical site infections, and urinary tract infections.

Propensity score matching (PSM) was not employed in this study because the primary objective was to explore predictors of revision surgery and its associated complications rather than to compare outcomes between matched groups. The well-established fact that revision surgeries generally result in higher complications compared to primary surgeries made PSM unnecessary for our analysis. Instead, multivariable logistic regression was chosen as it allows for adjustment of baseline covariates while maintaining the full sample size. This approach ensures the identification of independent predictors and associations without introducing bias from potential group imbalance. The potential bias from disproportionate group sizes is acknowledged as a limitation and discussed in the relevant section of the manuscript.

Survey weights provided by the NIS database were incorporated using the survey package in R to account for the complex sampling design and ensure nationally representative estimates. Each regression model was adjusted for patient demographics (age, sex, race), socioeconomic factors (primary payer), clinical characteristics (comorbidities including diabetes, hypertension, dyslipidemia, sleep apnea, anemia, alcohol abuse, mental disorders, Alzheimer’s disease, Parkinson’s disease, renal disease, chronic obstructive pulmonary disease, and heart failure), and hospital characteristics (region, bed size, and teaching status). All covariates except age were treated as categorical variables in the models. For categorical variables, appropriate reference categories were selected: White race for racial categories, Medicare for insurance status, and small bed size for hospital characteristics.

Odds ratios (ORs) with 95% confidence intervals (CIs) were calculated for each predictor variable. To account for the complex survey design of the NIS database, we employed the survey package in R, incorporating appropriate weights, strata, and cluster variables. All statistical analyses were conducted using R version 4.4.1. All statistical tests were two-tailed, and alpha levels ≤ 0.05 were considered statistically significant.

AI tools were employed exclusively to revise and enhance the clarity, grammar, and style of the English language in the manuscript. These tools were not utilized for data analysis, interpretation, or content generation.

## 3. Results

During 2016–2019, of the total cohort, 454,285 patients (~99.5%) underwent pLF, while 2470 patients (~0.5%) underwent revision procedures. Overall, the mean age of the cohort was 61.82 (12.39) years and 56.4% were women, mainly influenced by the majority of the patients who underwent the primary surgery. Table 2 presents the demographics and clinical characteristics for both primary and revision procedures. Patients undergoing rLF surgery were significantly younger compared to those undergoing pLF procedures (53.92 ± 20.65 vs. 61.87 ± 12.32 years, *p* < 0.001), with a comparable percentage of females (54.8% vs. 56.4%, *p* = 0.47). Racial distribution showed significant differences (*p* = 0.01), with a higher proportion of Black (10.5% vs. 7.9%) and Hispanic (7.5% vs. 5.6%) patients in the revision group and a lower proportion of White patients (73.7% vs. 79.4%) for this procedure. Insurance coverage also varied significantly (*p* < 0.001), with Medicaid coverage more prevalent among revision patients (12.6% vs. 6.1%) and Medicare coverage more common in the primary group (49.0% vs. 42.1%). Regarding comorbidities, revision patients exhibited significantly lower prevalence of hypertension (42.9% vs. 53.1%; *p* < 0.001) and dyslipidemia (31.8% vs. 40.3%; *p* < 0.001) but were otherwise similar to pLF patients across other conditions. Most surgeries were conducted at large hospitals, with revision procedures more likely to occur in medium and large hospitals (79.7% vs. 74.1%; *p* = 0.02). Both groups primarily had procedures at urban teaching hospitals, with a higher proportion in the revision group (77.1% vs. 72.6%; *p* = 0.04).

The various indications for these surgeries are shown in Table 3. Among these indications, spondylolisthesis was the most common etiology, accounting for 52.1% of the total cases, with a notable difference between primary (52.3%) and revision (8.5%) procedures (*p* < 0.001). Spinal stenosis was the second most prevalent condition, found in 31.9% of cases, comprising 32% of primary and 18.2% of revision surgeries.

### 3.1. Factors Associated with Revision Surgery

Based on the multivariate regression analysis comparing revision versus primary LF surgeries, several significant predictors were identified. Age demonstrated a protective effect, with each additional year associated with a 5% lower odds of revision surgery (OR 0.95, 95% CI 0.94–0.96, *p* < 0.001). Regarding insurance status, compared to Medicare (42.1%), patients with private insurance (OR 0.51, 95% CI 0.40–0.65, *p* < 0.001) and other insurance types (OR 0.59, 95% CI 0.41–0.84, *p* = 0.004) had significantly lower odds of undergoing revision surgery. Hospital characteristics also played a role, with both medium (OR 1.41, 95% CI 1.07–1.85, *p* = 0.01) and large bed size hospitals (OR 1.37, 95% CI 1.06–1.76, *p* = 0.01) associated with higher odds of performing revision surgeries compared to small hospitals. Other comorbidities, such as dyslipidemia (OR 1.01, 95% CI 0.82–1.25, *p* = 0.90) and hypertension (OR 0.88, 95% CI 0.73–1.05, *p* = 0.159), did not demonstrate significant associations with the likelihood of revision surgery.

### 3.2. Clinical Outcomes

Patients undergoing revision surgery demonstrated a higher overall complication rate (66.4% vs. 73.4%) compared to primary procedures. As shown in Table 4, revision surgeries were associated with significantly higher rates of surgical site infections (4.5% vs. 0.2%, OR 27.10, 95% CI 17.12–42.90, *p* < 0.001) and urinary tract infections (4.7% vs. 2.2%, OR 2.15, 95% CI 1.39–3.33, *p* < 0.001). Additionally, revision cases demonstrated increased likelihood of prolonged hospital stays (24.3% vs. 17.3%, OR 1.53, 95% CI 1.24–1.89, *p* < 0.001) but showed lower high-end hospital charges (17.9% vs. 25.0%, OR 0.65, 95% CI 0.51–0.83, *p* < 0.001). Other complications, including respiratory complications, dural tears, acute renal failure, thromboembolic events, blood loss anemia, and cardiac complications, showed no statistically significant differences between revision and primary procedures. In-hospital mortality rates were higher in revision cases but did not reach statistical significance (0.2% vs. 0.1%, OR 3.29, 95% CI 0.45–23.84, *p* = 0.23) (Table 4). In multivariate regression analysis, several significant predictors were identified for each major complication. For prolonged length of stay, revision surgery remained an independent risk factor (OR 1.54, 95% CI 1.24–1.92, *p* < 0.001), with alcohol abuse (OR 2.35, 95% CI 2.05–2.70, *p* < 0.001) and heart disease (OR 2.21, 95% CI 2.01–2.42, *p* < 0.001) showing the strongest associations. Both Alzheimer’s disease and chronic anemia demonstrated similar risk magnitudes (both OR 1.89, *p* < 0.001). Hospital teaching status (OR 1.53, 95% CI 1.44–1.63, *p* < 0.001) was also a significant predictor. Regarding high-end hospital charges, revision surgery was associated with lower odds (OR 0.59, 95% CI 0.46–0.76, *p* < 0.001). Geographic location showed significant impact, with the Western region demonstrating the highest odds (OR 3.48, 95% CI 2.88–4.21, *p* < 0.001). Hispanic ethnicity (OR 1.84, 95% CI 1.67–2.03, *p* < 0.001) and chronic anemia (OR 1.46, 95% CI 1.34–1.60, *p* < 0.001) were also associated with increased charges. Regarding the complication of surgical site infection, increased risk was most strongly associated with revision surgery (OR 24.25, 95% CI 14.89–39.48, *p* < 0.001), followed by congestive heart failure (OR 3.61, 95% CI 2.05–6.33, *p* < 0.001) and chronic anemia (OR 3.11, 95% CI 2.03–4.78, *p* < 0.001). Hispanic ethnicity showed increased risk (OR 2.23, 95% CI 1.36–3.63, *p* = 0.001), while female gender showed a protective effect (OR 0.64, 95% CI 0.48–0.86, *p* = 0.002). Regarding the complication of urinary tract infections, female gender was the strongest predictor (OR 2.79, 95% CI 2.50–3.11, *p* < 0.001) after revision surgery status (OR 2.42, 95% CI 1.54–3.79, *p* < 0.001). Significant comorbidity predictors included congestive heart failure (OR 1.67, 95% CI 1.35–2.05, *p* < 0.001), renal disease (OR 1.51, 95% CI 1.27–1.79, *p* < 0.001), and mental disorders (OR 1.33, 95% CI 1.21–1.46, *p* < 0.001). Age showed association with increased risk (OR 1.03 per year, 95% CI 1.02–1.03, *p* < 0.001).

**Figure 1:**
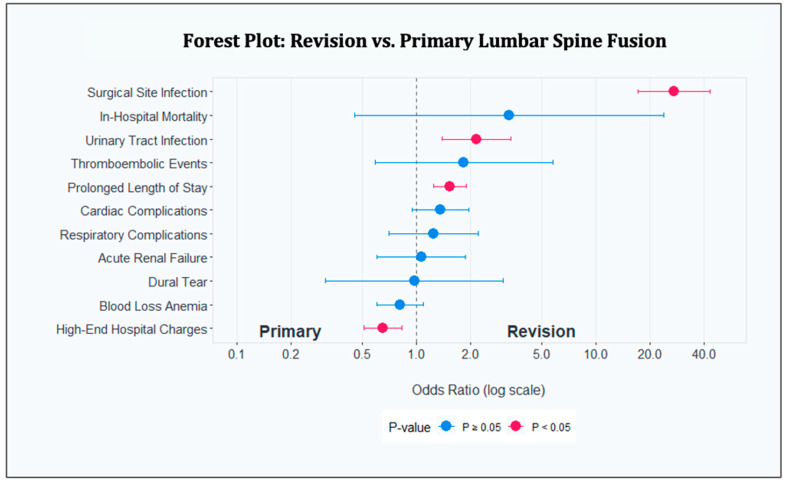
Forest plot of revision vs. primary lumbar spine fusion.

The forest plot (Figure 1) demonstrates these associations between outcomes and complications with the type of fusion procedure, with analyses adjusted for patient age, gender, race, primary payer, patient comorbidities, and hospital characteristics. Figure 1 shows the association of outcomes and complications with the type of fusion procedure (primary vs. revision). The outcomes and complications shown are the dependent variables used in our multivariable regression model, and the odds ratios represent the odds ratio of the exposure variable (type of fusion procedure, with primary being the reference value) for each of the respective regressions. Each outcome and complication were adjusted for patient age, gender, race, primary payer, patient comorbidities, and hospital characteristics.

Hospital outcomes demonstrate no significant difference in the median length of stay (3 days for both groups; *p* = 0.18), although revision patients incurred significantly lower median hospital charges compared to those undergoing primary fusion ($70,172 [$39,598–$128,069] vs. $104,965 [$70,919–$158,378]; *p* < 0.001) (Table 5).

## 4. Discussion

This study provides a comprehensive evaluation of the differences in outcomes and risk factors between primary and revision single-level lumbar fusion surgeries, offering important insights into the challenges of revision procedures. By leveraging a large, nationally representative database, this analysis highlights the increased complexity, higher complication rates, and distinct predictors associated with revision surgeries compared to primary procedures.

Revision surgeries are understandably more challenging than primary procedures, as they are associated with altered anatomy, scar tissue, and prior instrumentation, all contributing to increased technical difficulty and potential complications [10,11]. Given the variability in surgical approaches, patient selection, and hospital settings, benchmarking risk factors and outcomes is critical for identifying determinants of complications and improving patient-centered care. To this end, the National Inpatient Sample (NIS) provided a robust platform for evaluating nationwide trends and outcomes, representing 20% of all discharges across nonfederal U.S. hospitals [12].

Our analysis of 456,750 LF surgeries revealed significantly higher complication rates in revision procedures (66.4% vs. 73.4%), particularly surgical site infections and urinary tract infections. Several studies have identified risk factors for postoperative UTIs following lumbar spine fusion surgeries. Kurtz et al. reported a higher incidence of infections, including UTIs, in revision lumbar spine fusion procedures compared to primary procedures, with an adjusted hazard ratio (AHR) of 1.66 for revisions versus primary fusions. This increased risk is likely due to the greater complexity of revision surgeries, which often involve longer operative times and increased surgical trauma [13]. Similarly, Bohl et al. found that prolonged operative duration is a significant risk factor for UTIs following posterior lumbar fusion procedures, further highlighting the relevance of operative time in the increased risk observed in revision surgeries [14].

Notably, patients undergoing revision surgeries were significantly younger than those undergoing primary surgeries, potentially due to earlier onset of complications, greater biomechanical demands and higher level of expectations before their primary surgery [15,16,17] leading eventually to reoperation.

Regarding demographics and comorbidities, hypertension was the most prevalent comorbidity in both groups but was notably less common in revision patients (42.9% vs. 53.1%), consistent with their younger age profile. Similarly, revision patients showed lower rates of dyslipidemia and type 2 diabetes, suggesting a distinct comorbidity profile influenced by age. Racial and socioeconomic disparities were evident, with higher proportions of Black (10.5% vs. 7.9%) and Hispanic (7.5% vs. 5.6%) patients in the revision group, alongside greater Medicaid coverage (12.6% vs. 6.1%). These disparities raise important questions regarding access to initial care quality and align with recent literature highlighting inequities in spine surgery outcomes across racial and socioeconomic groups [18,19,20].

Regional and hospital-level differences were evident. Consistent with previous studies [9,19,20,21], lumbar fusions were predominantly performed in the South (40.9%), followed by the Midwest (23.3%) and West (20.4%). Urban teaching hospitals handled most surgeries, with a higher proportion of revisions (77.1%) performed in urban teaching hospitals compared to primary procedures (72.6%). These centers likely offer the specialized care required for complex revision cases, as patients undergoing lumbar fusion are at significantly higher risk for complications, including mortality and blood transfusions, particularly in revision surgeries [9].

Our multivariate regression analysis highlighted that revision patients are at increased risk for prolonged hospital stays, higher hospital charges, and complications such as neurologic issues, thromboembolic events, and infections. Specifically, revision surgeries were strongly associated with urinary tract infections (OR 2.79, 95% CI 2.50–3.11, *p* < 0.001) and surgical site infections (OR 24.25, 95% CI 14.89–39.48, *p* < 0.001). These findings are consistent with previous studies demonstrating higher odds of complications in revision surgeries [7,9,13,22]. The markedly higher risk of surgical site infections in revision cases (OR 24.25) suggests the need for enhanced perioperative protocols specifically tailored to revision procedures. This could include modified antibiotic prophylaxis regimens, specialized wound care protocols, and more intensive post-operative monitoring. In our study, we have found that inpatient mortality did not differ significantly between primary and revision surgeries, a reassuring finding despite the higher complication rates in revision procedures.

Interestingly, despite higher complication rates, revision procedures were associated with lower median hospital charges compared to primary procedures ($70,172 vs. $104,965, *p* < 0.001). This paradox may be attributable to differences in case complexity, resource allocation, or reimbursement structures. It is plausible that many of the revisions captured in the dataset involved less complex procedures, such as CSF leak repairs or wound debridement, which would inherently result in lower hospital charges compared to more extensive revision surgeries. Given the higher proportion of revisions performed in large centers, those findings emphasize the role of hospital expertise in revision cases. As Francis et al. noted, disparities between rural and urban settings may also play a role, reflecting differences in healthcare access, cultural factors, and resource utilization [18,19,23].

Understanding baseline risks for adverse outcomes in revision surgeries can inform presurgical evaluation, risk stratification, and shared decision-making. Evaluating complication rates as highlighted in our analysis could serve as an adjunct for patient counseling, offering evidence-based guidance on treatment options and expected outcomes. The association of chronic conditions with adverse outcomes emphasizes the need for multidisciplinary care pathways to manage comorbidities effectively.

Prophylactic strategies, such as anticoagulation for thromboembolic prevention, along with optimized surgical techniques and robust postoperative protocols, are essential for mitigating complications in revision surgeries. However, our findings warrant deeper investigation into the socioeconomic factors affecting surgical outcomes. This investigation could enable two critical improvements: first, enhancing conservative treatment approaches and delaying primary surgery timing, thereby lowering the odds for revision surgery; and second, refining patient selection criteria to achieve better clinical outcomes and reduce the likelihood of requiring revision procedures. This comprehensive approach—addressing both immediate clinical concerns and underlying socioeconomic factors—is crucial for fostering equity in lumbar fusion management while systematically reducing the need for revision surgeries across all patient populations. 

### Limitations

This study has several limitations inherent to retrospective database analyses. The reliance on ICD-10 codes introduces potential for misclassification and coding errors, for instance, our analysis was unable to determine whether revision surgeries were specifically performed for Adjacent Segment Disease (ASD) due to the absence of specific ICD-10 codes identifying ASD as the etiology for revision. Additionally, the observational nature of the study limits causal inference between predictors and outcomes. The use of de-identified data precludes the assessment of long-term outcomes beyond hospital discharge. While the NIS dataset provides a nationally representative sample, it lacks granular details on surgeon expertise, operative techniques, and institutional protocols, all of which could influence outcomes. One of the primary limitations of this study stems from the use of the ICD-10 Procedure Coding System, which lacks information regarding the specific levels of the spine involved in primary or revision lumbar fusion surgeries. Consequently, it is not feasible to identify or compare the levels of lumbar fusion using the data from the NIS database. Additionally, this coding system does not provide detailed information about the types of instrumentation utilized during spinal fusion procedures. This limitation precludes a comparative analysis of outcomes based on different instrumentation techniques. 

Another limitation of our study is the significant disparity in sample sizes between the primary surgery group and the revision surgery group, with the latter comprising only a small fraction of the total sample (0.5%). This imbalance may introduce bias in the statistical analyses and limit the precision of estimates for the revision group. Although multivariable logistic regression was used to adjust for confounders, the small size of the revision group reduces the robustness of findings specific to revision surgeries.

Additionally, the increasing trend toward outpatient LF may result in underestimates of the true number of procedures performed during the study period. Although our study leverages a large sample size, providing robust statistical power and generalizability, it lacks the detailed clinical data and longitudinal follow-up available in randomized controlled trials. Future studies incorporating longitudinal data and patient-reported outcomes are warranted to provide a more comprehensive understanding of the long-term impact of revision surgeries.

## 5. Conclusions

This study demonstrates distinct differences in demographics, clinical characteristics, and outcomes between primary and revision single-level lumbar fusion surgeries. Revision procedures are associated with higher complication rates but lower charges and shorter hospital stays. Identifying and addressing risk factors for revision surgery and associated complications remains critical. Future studies should explore the role of surgeon and hospital-level factors, as well as long-term patient-reported outcomes, to provide a more holistic understanding of lumbar fusion surgery care.

## Figures and Tables

**Table 1:** ICD-10 diagnostic and procedure codes used to define study variables.

Category	Subcategory	ICD10_Codes
Procedures	Primary Lumbar Fusion	0SG0070, 0SG0071, 0G007J, 0SG00A0, 0SG00AJ, 0SG00J0, 0SG00J1, 0SG00JJ, 0SG00K0, 0SG00K1, 0SG0370, 0SG0371, 0SG037J, 0SG03A0, 0SG03AJ, 0SG03J0, 0SG03J1, 0SG03K0, 0SG03K1, 0SG03KJ, 0SG0471, 0SG047J, 0SG04A0, 0SG04AJ, 0SG04J0, 0SG04J1, 0SG04K1, 0SG04KJ
	Revision Lumbar Surgery	0SW00AZ, 0SW03AZ, 0SW04AZ, 0SW0XAZ, 0QW00JZ, 0QW03JZ, 0QW0XJZ, 0SW008Z, 0SW038Z, 0SW0X8Z, 0RW00AZ, 0RW03AZ, 0RW04AZ, 0RW0XAZ, 0RW10AZ, 0RW13AZ, 0RW14AZ, 0RW1XAZ, 0RW40AZ, 0RW43AZ, 0RW44AZ, 0RW4XAZ, 0RW60AZ, 0RW63AZ, 0RW64AZ, 0RW6XAZ, 0RWA0AZ, 0RWA3AZ, 0RWA4AZ, 0RWAXAZ, 0SW30AZ, 0SW33AZ, 0SW34AZ, 0SW3XAZ, 0QW004Z, 0QW007Z, 0QW00KZ, 0QW034Z, 0QW037Z, 0QW03KZ, 0QW044Z, 0QW047Z, 0QW04JZ, 0QW04KZ, 0QW0X4Z, 0QW0X7Z, 0QW0XKZ, 0SW000Z, 0SW003Z, 0SW004Z, 0SW007Z, 0SW00JZ, 0SW00KZ, 0SW030Z, 0SW033Z, 0SW034Z, 0SW037Z, 0SW03JZ, 0SW03KZ, 0SW040Z, 0SW043Z, 0SW044Z, 0SW047Z, 0SW048Z, 0SW04JZ, 0SW04KZ, 0SW0X0Z, 0SW0X3Z, 0SW0X4Z, 0SW0X7Z, 0SW0XJZ, 0SW0XKZ, 0SW200Z, 0SW203Z, 0SW207Z, 0SW20JZ, 0SW20KZ, 0SW230Z, 0SW233Z, 0SW237Z, 0SW23JZ, 0SW23KZ, 0SW240Z, 0SW243Z, 0SW247Z, 0SW24JZ, 0SW24KZ, 0SW2X0Z, 0SW2X3Z, 0SW2X7Z, 0SW2XJZ, 0SW2XKZ
Surgical Etiology	Spondylolisthesis	M4316
	Spinal Stenosis	M4806
	Disk Degeneration	M5136
	Spondylosis	M47896
	Radiculopathy	M5416
	Scoliosis	M4186
	Spinal Instabilities	M532X6
	Spondylolysis	M4306
Comorbidities	Type 2 Diabetes	E11 *
	Hypertension	I10
	Dyslipidemia	E78 *
	Obstructive Sleep Apnea	G473 *
	Anemia	D64 *
	Alcohol Abuse	F10 *
	Mental Disorders	F *
	Alzheimer’s Disease	G30 *
	Parkinson’s Disease	G20
	Chronic Kidney Disease	N18 *
	Chronic Obstructive Pulmonary Disease	J44 *
	Heart Failure	I50 *
Complications	Surgical Site Infection	T814 *
	Urinary Tract Infection	N39 *
	Pneumonia	J18 *, J15 *, J22
	Respiratory Failure	J96 *
	Cardiac Arrest	I46 *
	Myocardial Infarction	I20-24 *
	Acute Renal Failure	N17 *
	Embolism (DVT, PE)	I2602, I2609, I2692, I2699, I82401-I82429
	Acute Blood Loss	D62 *
	Nerve Root Injury/CSF Leak	G9611, G9600, G9602, S34 *
* Starts with		

**Table 2:** Demographics and patient characteristics among the primary and revision lumbar fusion groups.

	Overall (n = 456,750)	Primary (n = 454,280)	Revision (n = 2470)	*p*-Value
**Age (mean (SD))**	61.82 (12.39)	61.87 (12.32)	53.92 (20.65)	<0.001
**Female, n (%)**	257,490 (56.4)	256,140 (56.4)	1350 (54.8)	0.47
**Race, n (%)**				0.01
White	362,390 (79.3)	360,570 (79.4)	1820 (73.7)	
Black	36,080 (7.9)	35,820 (7.9)	260 (10.5)	
Hispanic	25,600 (5.6)	25,415 (5.6)	185 (7.5)	
Asian	5630 (1.2)	5610 (1.2)	20 (0.8)	
Other	27,050 (5.9)	26,865 (5.9)	185 (7.5)	
**Primary Payer, n (%)**				<0.001
Medicare	223,540 (48.9)	222,500 (49)	1040 (42.1)	
Medicaid	28,070 (6.1)	27,760 (6.1)	310 (12.6)	
Private	170,925 (37.4)	170,035 (37.4)	890 (36.0)	
Other	34,215 (7.5)	33,985 (7.5)	230 (9.3)	
**Comorbidities, n (%)**				
Type 2 DM	99,835 (21.9)	99,360 (21.9)	475 (19.2)	0.14
Hypertension	242,250 (53.0)	241,190 (53.1)	1060 (42.9)	<0.001
Dyslipidemia	183,910 (40.3)	183,125 (40.3)	785 (31.8)	<0.001
Obstructive Sleep Apnea	70,165 (15.4)	69,840 (15.4)	325 (13.2)	0.17
Chronic Anemia	22,485 (4.9)	22,345 (4.9)	140 (5.7)	0.44
Alcohol Abuse	5055 (1.1)	5045 (1.1)	10 (0.4)	0.13
Mental Disorders	175,815 (38.5)	174,905 (38.5)	910 (36.8)	0.44
Alzheimer’s Disease	555 (0.1)	555 (0.1)	0	0.44
Parkinson’s Disease	4015 (0.9)	3990 (0.9)	25 (1)	0.75
Chronic Renal Disease	27,420 (6.0)	27,315 (6.0)	105 (4.3)	0.10
COPD	35,040 (7.7)	34,835 (7.7)	205 (8.3)	0.59
CHF	11,820 (2.6)	11,730 (2.6)	90 (3.6)	0.13
**Hospital Characteristics**				
Hospital Bed Size, n (%)				0.02
Small	118,220 (25.9)	117,720 (25.9)	500 (20.2)	
Medium	120,635 (26.4)	119,925 (26.4)	710 (28.7)	
Large	217,895 (47.7)	216,635 (47.7)	1260 (51)	
Hospital Location/Teaching, n (%)				0.04
Rural	17,555 (3.8)	17,450 (3.8)	105 (4.3)	
Urban Non-Teaching	107,645 (23.6)	107,185 (23.6)	460 (18.6)	
Urban Teaching	331,550 (72.6)	329,645 (72.6)	1905 (77.1)	
Hospital Region, n (%)				0.42
Northeast	70,280 (15.4)	69,910 (15.4)	370 (15)	
Midwest	106,380 (23.3)	105,770 (23.3)	610 (24.7)	
South	186,870 (40.9)	185,810 (40.9)	1060 (42.9)	
West	93,220 (20.4)	92,790 (20.4)	430 (17.4)	

**Table 3:** Patients’ primary indication for LF surgery by surgery type.

	Overall (n = 456,750)	Primary (n = 454,280)	Revision (n = 2470)	*p*-Value
**Etiology, n (%)**				<0.001
Spondylolisthesis	237,755 (52.1)	237,545 (52.3)	210 (8.5)	
Spinal stenosis	145,775 (31.9)	145,325 (32)	450 (18.2)	
Disk degeneration	10,335 (2.3)	10,285 (2.3)	50 (2)	
Spondylosis	1710 (0.4)	1695 (0.4)	15 (0.6)	
Radiculopathy	5665 (1.2)	5430 (1.2)	235 (9.5)	
Scoliosis	905 (0.2)	900 (0.2)	5 (0.2)	
Spinal instabilities	2735 (0.6)	2730 (0.6)	5 (0.2)	
Spondylolysis	620 (0.1)	610 (0.1)	10 (0.4)	

**Table 4:** Summary of complications, in-hospital mortality, and hospital outcomes among primary lumbar fusion vs. revision group.

	Primary (n = 454,280)	Revision (n = 2470)	Odds Ratio (95% CI)	*p*-Value
**Complications, n (%)**	66.4	73.4		
Surgical Site Infection	780 (0.2)	110 (4.5)	27.10 (17.12–42.90)	**<0.001**
Urinary Tract Infection	10.73 (2.2)	115 (4.7)	2.15 (1.39–3.33)	**<0.001**
Respiratory Complications	8930 (2)	60 (2.4)	0.85 (0.68–1.06)	0.14
Dural Tear, CSF leak	2845 (0.6)	15 (0.6)	1.24 (0.70–2.19)	0.45
Acute Renal Failure	10,390 (2.3)	60 (2.4)	0.97 (0.31–3.02)	0.95
Embolism (DVT, PE)	1510 (0.3)	15 (0.6)	1.06 (0.60–1.87)	0.83
Blood Loss Anemia	54,530 (12.0)	245 (9.9)	1.83 (0.59–5.73)	0.29
Cardiac Complications	19,960 (4.4)	145 (5.9)	1.36 (0.94–1.96)	0.10
**Hospital Outcomes**				
Prolonged Length of Stay	78,545 (17.3)	600 (24.3)	1.53 (1.24–1.89)	**<0.001**
High-End Hospital Charges	113,330 (25)	440 (17.9)	0.65 (0.51–0.83)	**<0.001**
**Hospital Outcomes**				
Prolonged Length of Stay	78,545 (17.3)	600 (24.3)	1.53 (1.24–1.89)	**<0.001**
High-End Hospital Charges	113,330 (25)	440 (17.9)	0.65 (0.51–0.83)	**<0.001**
In-Hospital Mortality	280 (0.1)	5 (0.2)	3.29 (0.45–23.84)	0.23

Bold *p*-values indicate statistical significance at α = 0.05. DVT, deep vein thrombosis; PE, pulmonary embolism; CSF, cerebrospinal fluid.

**Table 5:** Comparison of hospitalization outcomes among the primary vs. the revision lumbar fusion cohorts.

	Overall (n = 456,750)	Primary LF (n = 454,280)	Revision LF (n = 2470)	*p*-Value
**Total Charges ($)**	104,792 [70,745.02–158,223.02]	104,965 [70,919–158,378]	70,172 [39,598–128,069]	<0.001
**Length of Stay (days)**	3 [2–4]	3 [2–4]	3 [1–4]	0.18

## Data Availability

Restrictions apply to the availability of these data. Data were obtained from HCUP and are available [https://hcup-us.ahrq.gov/] with the permission of HCUP.

## List of Abbreviations

HCUP	Healthcare Cost and Utilization Project
ICD-10	International Classification of Diseases, 10th Revision
LF	Lumbar Fusion
LOS	Length of Stay
NIS	Nationwide Inpatient Sample
pLF	Primary Lumbar Fusion
rLF	Revision Lumbar Fusion
SPSS	Statistical Package for the Social Sciences
